# Supplementary material for: Time‑resolved multi-omic analysis of paclitaxel exposure in human iPSC‑derived sensory neurons unveils mechanisms of chemotherapy‑induced peripheral neuropathy
Source: Cell Death Dis. 2026 Feb 10;17(1):211. doi: 10.1038/s41419-026-08445-2 (PMC12921266; doi:10.1038/s41419-026-08445-2)
Supplement: Supplementary file 15 — Dataset 1. [file 41419_2026_8445_MOESM15_ESM.zip › CDD_Open_Data/04_RNA_Seq/Timeline_panel_plots_etc_2023-01-23_1609.zip!/Panel_Plots_etc/Evidence_Plots/post5d/evidence_plot_standard contrasts_msigdb_reactome_5d_PTX_vs_DMSO_ID6_M752_REACTOME_NEUROTRANSMITTER_RECEPTORS_AND_POSTSYNAPTIC_SIGNAL_TRANSMISSION.pdf]

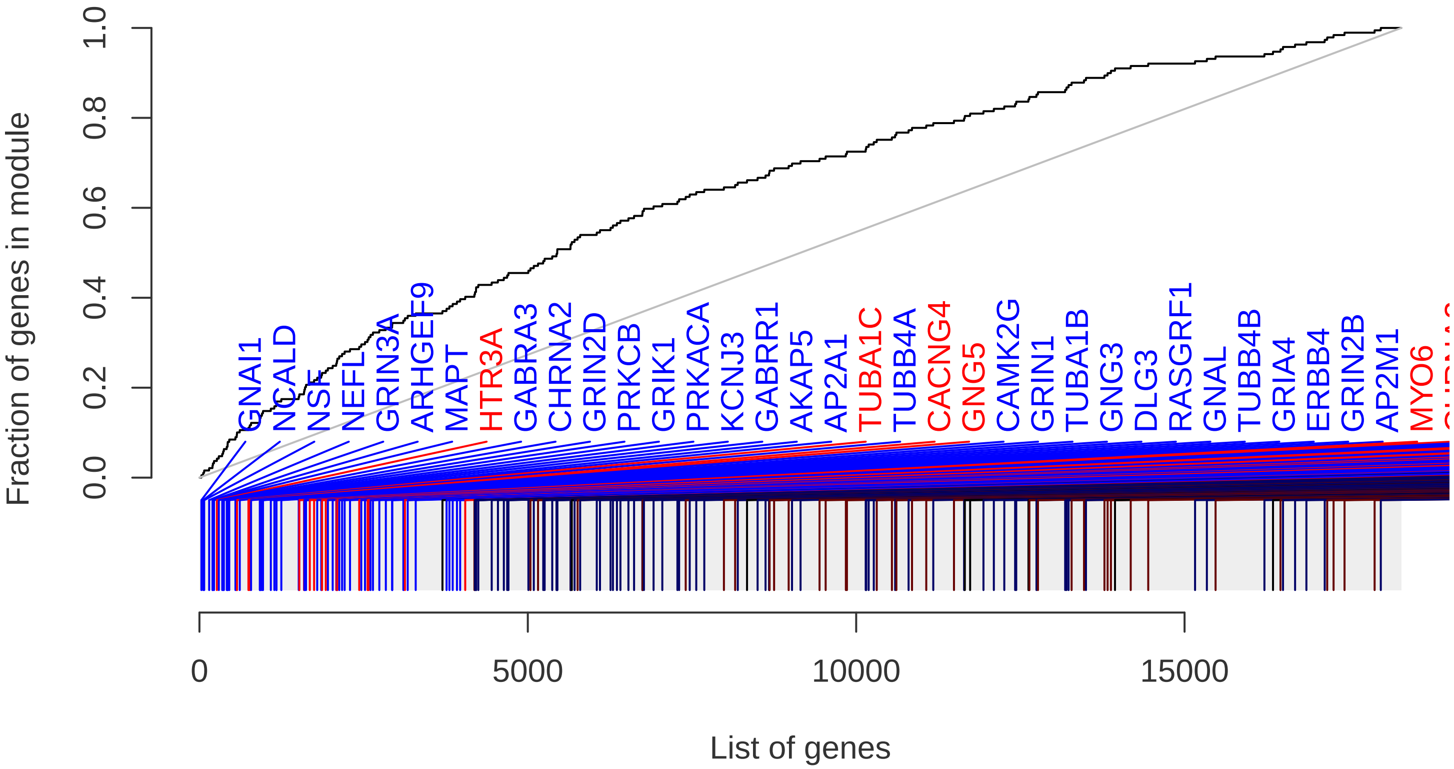
Fraction of genes in module
0.0
0.2
0.4
0.6
0.8
1.0
GNAI1
NCALD
NSF
NEFL
GRIN3A
ARHGEF9
MAPT
HTR3A
GABRA3
CHRNA2
GRIN2D
PRKCB
GRIK1
PRKACA
KCNJ3
GABRR1
AKAP5
AP2A1
TUBA1C
TUBB4A
CACNG4
GNG5
CAMK2G
GRIN1
TUBA1B
GNG3
DLG3
RASGRF1
GNAL
TUBB4B
GRIA4
ERBB4
GRIN2B
AP2M1
MYO6
0
5000
10000
15000
List of genes
